# Supplementary material for: Resolving the full spectrum of human genome variation using Linked-Reads
Source: Genome Res. 2019 Apr;29(4):635–45. doi: 10.1101/gr.234443.118 (PMC6442396; doi:10.1101/gr.234443.118)
Supplement: Supplemental Material [file supp_gr.234443.118_Supplemental_longranger-2.2.zip › longranger-2.2.tar.bz2!/lr2.2-public/lib/stan/cmdstan/stan/doc/api/html/math_2functions_8hpp_source.html]

Stan: src/stan/math/functions.hpp Source File


|  |  |
| --- | --- |
|  | Stan  2.5.0  probability, sampling & optimization |


- Main Page
- Related Pages
- Namespaces
- Classes
- Files

- File List
- File Members

All Classes Namespaces Files Functions Variables Typedefs Enumerations Enumerator Friends Macros Pages

- src
- stan
- math

functions.hpp

Go to the documentation of this file.

1 #ifndef STAN\_\_MATH\_\_FUNCTIONS\_HPP

2 #define STAN\_\_MATH\_\_FUNCTIONS\_HPP

3

4 #include <stan/math/functions/Phi.hpp>

5 #include <stan/math/functions/Phi\_approx.hpp>

6 #include <stan/math/functions/abs.hpp>

7 #include <stan/math/functions/as\_bool.hpp>

8 #include <stan/math/functions/bessel\_first\_kind.hpp>

9 #include <stan/math/functions/bessel\_second\_kind.hpp>

10 #include <stan/math/functions/binary\_log\_loss.hpp>

11 #include <stan/math/functions/binomial\_coefficient\_log.hpp>

12 #include <stan/math/functions/digamma.hpp>

13 #include <stan/math/functions/dist.hpp>

14 #include <stan/math/functions/divide.hpp>

15 #include <stan/math/functions/dot.hpp>

16 #include <stan/math/functions/dot\_self.hpp>

17 #include <stan/math/functions/exp2.hpp>

18 #include <stan/math/functions/falling\_factorial.hpp>

19 #include <stan/math/functions/fdim.hpp>

20 #include <stan/math/functions/gamma\_p.hpp>

21 #include <stan/math/functions/gamma\_q.hpp>

22 #include <stan/math/functions/ibeta.hpp>

23 #include <stan/math/functions/if\_else.hpp>

24 #include <stan/math/functions/int\_step.hpp>

25 #include <stan/math/functions/inv.hpp>

26 #include <stan/math/functions/inv\_cloglog.hpp>

27 #include <stan/math/functions/inv\_logit.hpp>

28 #include <stan/math/functions/inv\_sqrt.hpp>

29 #include <stan/math/functions/inv\_square.hpp>

30 #include <stan/math/functions/inverse\_softmax.hpp>

31 #include <stan/math/functions/is\_inf.hpp>

32 #include <stan/math/functions/is\_nan.hpp>

33 #include <stan/math/functions/is\_uninitialized.hpp>

34 #include <stan/math/functions/lbeta.hpp>

35 #include <stan/math/functions/lgamma.hpp>

36 #include <stan/math/functions/lmgamma.hpp>

37 #include <stan/math/functions/log1m.hpp>

38 #include <stan/math/functions/log1m\_exp.hpp>

39 #include <stan/math/functions/log1m\_inv\_logit.hpp>

40 #include <stan/math/functions/log1p.hpp>

41 #include <stan/math/functions/log1p\_exp.hpp>

42 #include <stan/math/functions/log2.hpp>

43 #include <stan/math/functions/log\_diff\_exp.hpp>

44 #include <stan/math/functions/log\_falling\_factorial.hpp>

45 #include <stan/math/functions/log\_inv\_logit.hpp>

46 #include <stan/math/functions/log\_rising\_factorial.hpp>

47 #include <stan/math/functions/log\_sum\_exp.hpp>

48 #include <stan/math/functions/logical\_and.hpp>

49 #include <stan/math/functions/logical\_eq.hpp>

50 #include <stan/math/functions/logical\_gt.hpp>

51 #include <stan/math/functions/logical\_gte.hpp>

52 #include <stan/math/functions/logical\_lt.hpp>

53 #include <stan/math/functions/logical\_lte.hpp>

54 #include <stan/math/functions/logical\_negation.hpp>

55 #include <stan/math/functions/logical\_neq.hpp>

56 #include <stan/math/functions/logical\_or.hpp>

57 #include <stan/math/functions/logit.hpp>

58 #include <stan/math/functions/max.hpp>

59 #include <stan/math/functions/min.hpp>

60 #include <stan/math/functions/modified\_bessel\_first\_kind.hpp>

61 #include <stan/math/functions/modified\_bessel\_second\_kind.hpp>

62 #include <stan/math/functions/modulus.hpp>

63 #include <stan/math/functions/multiply\_log.hpp>

64 #include <stan/math/functions/owens\_t.hpp>

65 #include <stan/math/functions/primitive\_value.hpp>

66 #include <stan/math/functions/rising\_factorial.hpp>

67 #include <stan/math/functions/scaled\_add.hpp>

68 #include <stan/math/functions/square.hpp>

69 #include <stan/math/functions/step.hpp>

70 #include <stan/math/functions/sub.hpp>

71 #include <stan/math/functions/sum.hpp>

72 #include <stan/math/functions/trigamma.hpp>

73 #include <stan/math/functions/value\_of.hpp>

74

75 #endif

log\_sum\_exp.hpp

rising\_factorial.hpp

dist.hpp

modulus.hpp

log\_rising\_factorial.hpp

multiply\_log.hpp

log1m\_exp.hpp

digamma.hpp

min.hpp

log1m\_inv\_logit.hpp

falling\_factorial.hpp

gamma\_q.hpp

log1p\_exp.hpp

Phi\_approx.hpp

inverse\_softmax.hpp

logit.hpp

lmgamma.hpp

Phi.hpp

square.hpp

logical\_and.hpp

modified\_bessel\_first\_kind.hpp

as\_bool.hpp

exp2.hpp

logical\_negation.hpp

sum.hpp

log2.hpp

dot\_self.hpp

bessel\_second\_kind.hpp

ibeta.hpp

logical\_lte.hpp

divide.hpp

dot.hpp

logical\_neq.hpp

log\_diff\_exp.hpp

log1m.hpp

fdim.hpp

inv.hpp

logical\_gt.hpp

sub.hpp

logical\_or.hpp

if\_else.hpp

step.hpp

log\_inv\_logit.hpp

log\_falling\_factorial.hpp

logical\_gte.hpp

trigamma.hpp

gamma\_p.hpp

logical\_eq.hpp

binary\_log\_loss.hpp

primitive\_value.hpp

owens\_t.hpp

log1p.hpp

inv\_square.hpp

inv\_cloglog.hpp

abs.hpp

scaled\_add.hpp

is\_nan.hpp

int\_step.hpp

bessel\_first\_kind.hpp

value\_of.hpp

inv\_logit.hpp

inv\_sqrt.hpp

binomial\_coefficient\_log.hpp

logical\_lt.hpp

lbeta.hpp

modified\_bessel\_second\_kind.hpp

is\_uninitialized.hpp

lgamma.hpp

max.hpp

is\_inf.hpp


---

[ Stan Home Page ]

*© 2011–2014,
Stan Development Team.*
